# A Cold-Blooded View on Adult Neurogenesis

**DOI:** 10.3389/fnins.2017.00327

**Published:** 2017-06-08

**Authors:** Anabel R. Simões, Christa Rhiner

**Affiliations:** Champalimaud Centre for the UnknownLisbon, Portugal

**Keywords:** adult neurogenesis in arthropods, *Drosophila*, adult neural stem cells, regenerative neurogenesis, activation of quiescent stem cells

## Abstract

During brain development, highly complex and interconnected neural circuits are established. This intricate wiring needs to be robust to faithfully perform adult brain function throughout life, but at the same time offer room for plasticity to integrate new information. In the mammalian brain, adult-born neurons are produced in restricted niches harboring neural stem cells. In the fruit fly *Drosophila*, low-level adult neurogenesis arising from a dispersed population of neural progenitors has recently been detected in the optic lobes. Strikingly, these normally quiescent neural stem cells proliferate upon brain injury and produce new neurons for brain regeneration. Here, we review adult neurogenesis in crustaceans and insects and highlight that neurogenesis in the visual system is prominent in arthropods, but its role and underlying mechanisms are unclear. Moreover, we discuss how the study of damage-responsive progenitor cells in *Drosophila* may help to understand robust regenerative neurogenesis and open new avenues to enhance brain repair after injury or stroke in humans.

The generation and integration of new neurons into the adult brain based on quiescent adult neural stem cells is a conserved trait in mammals (Kempermann, 2012). In the rodent brain, where most research has concentrated on so far, two canonical regions of adult neurogenesis exist (Zhao C. et al., 2008). First, the subventricular zone of the lateral ventricle, which produces neuroblasts that will migrate into the olfactory bulb to form new olfactory interneurons and second, the hippocampus, where adult-born neurons are thought to contribute to learning and memory (Bond et al., 2015; Gonçalves et al., 2017).

This review will focus on adult neurogenesis in crustaceans and insects, which have recently gained more protagonism in the field and show the potential to offer new mechanistic insight into aspects of stem cell activation, regeneration, and evolutionary conserved functions of newborn neurons in the mature brain.

## Adult neurogenesis in crustaceans

**Figure 1:**
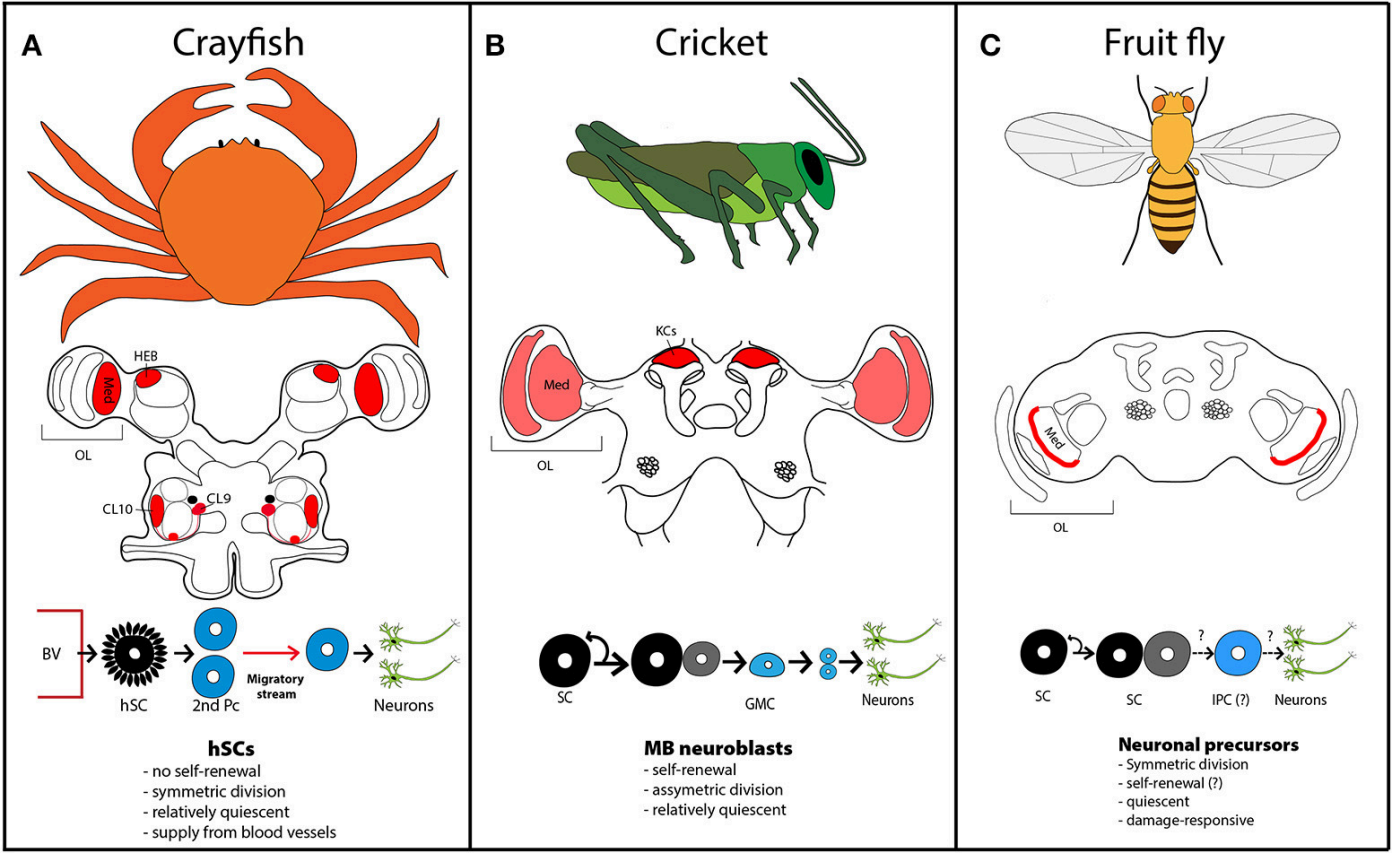
Neurogenic zones in crustacean and insect species. **(A)** Adult neurogenesis in crayfish occurs in the medulla (med) of the optic lobe (OL), the hemiellipsoid body (HEB) and in cluster (CL) 9 and 10 adjacent to the olfactory lobe. The red circle below the clusters designates the niche. Circulating hemocytes in the blood enter the niche in the brain via underlying blood vessels (BVs). The progeny of these hemocyte-derived stem cells (hSCs), called secondary precursors (2nd Pcs) migrate along streams to the proliferation clusters, where they divide and differentiate into olfactory interneurons. **(B)** In crickets, adult-proliferating cells are found in the optic lobes and the cortex of the adult mushroom bodies. New neurons arise from mushroom body (MB) neuroblasts, which divide asymmetrically to self-renew and produce a ganglion mother cell (GMC), which in turn divides to form two neurons. KC, Kenyon cells. **(C)** Low-level adult neurogenesis occurs in the outer medulla of fruit fly. Adult-born neurons derive from symmetric divisions of neural precursors and likely intermediate proliferating cells (IPCs). Neurogenic zones are shaded in red. Light red indicates proliferating zones, where no published images are available.

Arthropods represent the most species-rich phylum, comprising myriapods (e.g., centipedes), the extinct trilobites, the chelicerates (e.g., spiders), crustaceans, and insects, whereas the latter two subphyla are more closely related. Adult neurogenesis is well-documented in numerous species of crustaceans. In decapod crustaceans, such as lobster and crayfish, the formation and integration of adult-born neurons has been especially well-studied (Harzsch and Dawirs, 1996; Schmidt, 1997, 2001, 2007; Harzsch et al., 1999a; Sullivan and Beltz, 2005). These aquatic invertebrates are long-lived and show indeterminate growth during adulthood. Since they keep growing as adults, their brain needs to integrate an ever-increasing amount of sensory input from new sensilla on the body and from ommatidia of the eye. Strikingly, new neurons are only incorporated in few brain areas, mainly the medulla of the optic lobe (Schmidt, 1997; Sullivan and Beltz, 2005) and two clusters associated with the olfactory and accessory lobes (Schmidt and Harzsch, 1999; Beltz and Sandeman, 2003; Figure 1A). In contrast, additional afferents from chemo- and mechanosensory neurons are integrated via enhanced arborizations and connectivity of preexisting neurons (Schmidt, 2007).

## Adult neurogenesis sustained by immigrants

What is the source of new neurons in the crustacean brain? By now, the entire cellular path leading to adult neurogenesis in freshwater crayfish has been discovered (Benton et al., 2011, 2014): Similar to mammals, the brain of crayfish contains a neurogenic niche formed by glial cells, which harbors neural progenitor cells. However, these first-generation precursors are not self-renewing (Benton et al., 2011); they divide symmetrically and both daughter cells, termed second-generation precursors, migrate along fibrous streams to proliferation zones near the olfactory lobe, where they divide at least once more before their progeny differentiates into olfactory interneurons or projection neurons (Sullivan and Beltz, 2005; Kim et al., 2014; Figure 1A).

Since the precursors in the niche do not self-renew, there must be a way to replenish the niche to sustain life-long neurogenesis. Recently, Barbara Beltz and group showed that circulating hemocytes (blood cells), which can access the neurogenic niche via the cerebral artery (Chaves da Silva et al., 2013), are a source of first-generation neural precursors (Benton et al., 2014). When they injected labeled hemocytes from a donor crayfish into a recipient animal, these immune cells efficiently homed to the neurogenic niche—an attraction mediated partly by serotonin signaling (Benton et al., 2011)—and gave rise to second generation precursors and ultimately differentiated neurons (Benton et al., 2014). This shows that in crayfish, cells outside the brain are responsible of regulating adult neurogenesis. In contrast, neurogenesis during development is based—very much like in insects—on asymmetric divisions of classic neuroblasts (Harzsch et al., 1999b).

What is the function of neurogenesis in these particular regions? Obviously, neurogenesis is not just a compensatory reaction to accommodate more sensory input in growing crustaceans, as discussed above. In the olfactory system, the integration of new neurons may lead to increased combinatorial coding and therefore the ability to distinguish new odors. This could have a survival advantage for long-lived crustaceans, which often migrate over long distances and change habitat (Schmidt, 2007). In rodents, olfactory bulb neurogenesis seems to be important for efficient odor discrimination learning (Alonso et al., 2012), but also a function in mate choice is likely (Mak et al., 2007). Olfactory neurogenesis may therefore represent a conserved trait to allow olfactory fine-tuning in long-lived animals, given that the maturation of adult-born neurons takes several weeks to months.

Overall, neurogenesis in the olfactory pathway of crayfish shows many parallels to adult neurogenesis in the olfactory bulb of rodents, starting from the slow-dividing progenitors (≥48 h) (Benton et al., 2011) to the migratory stream of neuroblasts and their differentiation into olfactory interneurons (Figure 1A). On the other hand, neural precursors in crustaceans do not seem to form glial lineages compared to mammalian adult neural stem cells and they are continuously refreshed from hemocytes since they lack self-renewing stem cell properties (Benton et al., 2014).

## The optic lobe as neurogenic zone

In the small crab and crayfish, adult neurogenesis occurs in the optic lobe, but has not been investigated in detail. Proliferating cells have been observed in several zones of the adult optic lobe, but evidence that they differentiate into neurons has only been obtained for the medulla externa (Sullivan and Beltz, 2005; Figure 1A). If new neurons were required to maintain the highly columnar organization of the visual pathway, one would also expect to find adult-born neurons in the lamina. However, it might be very challenging to understand the functions of new neurons in visual circuits of crustaceans because they have very complex eyes. There are species like the mantis shrimp, which show up to 16 different photoreceptors and trinocular vision (Thoen et al., 2014).

Finally, the addition of local interneurons has also been observed in the hemiellipsoid body of the shore crab (Schmidt, 1997). The hemiellipsoid body is located close to the optic lobes and may be homologous to the mushroom bodies of insects, which are higher brain centers required for memory formation. This raises the question if neurogenesis in crustaceans could also be important for orientation or learning. Given that adult neurogenesis in crayfish can be suppressed by reducing peripheral hemocytes, without manipulating the nervous system, it will be interesting to see how altered neurogenesis impacts on behavior.

## Regeneration in the crustacean brain

In crayfish, the overall level of neurogenesis is regulated by the number of circulating peripheral hemocytes (Benton et al., 2014). This strong link between the immune system and a stem cell niche is remarkable and could represent an efficient mechanism to directly convert immune cell activation into stem cell proliferation and regeneration in case of inflammation and tissue damage.

Regeneration in decapod crustaceans has been described since the 18th century for the appendages and for the compound eye (Steele, 1907; Cooper, 1998). Several species of crustaceans have the ability to regenerate the ommatidia of the eye after partial removal of the cornea, like the hermit crab and several shrimp and crayfish species. Hermit crabs can even regenerate a perfect eye after removal of the cornea and half of the optic ganglion (Steele, 1907). These experiments, together with the observed adult neurogenesis in optic lobes, suggest that neural precursors also migrate to and proliferate in the optic ganglia (Steele, 1907). Now that the cellular basis of adult neurogenesis is known in crayfish, it would be interesting to study its role and regulation in eye and optic ganglion regeneration.

## Adult neurogenesis in insects

In insects, adult neurogenesis is known to occur in two different regions: the mushroom bodies (MBs) and the optic lobes (Cayre et al., 1996; Figure 1B). Strikingly, almost all studies have concentrated on MBs, leaving optic lobe neurogenesis virtually unexplored. Therefore, we will first consider findings in MBs, before shifting the discussion to the visual system.

The MBs represent higher brain centers for multimodal sensory integration in the protocerebrum of insects (Farris and Sinakevitch, 2003). They consist of hundreds to hundreds of thousands densely packed interneurons, called Kenyon cells (KCs), and a neuropil formed by their projections and synaptic contacts. The MBs receive input from the olfactory and gustatory system, in hymenoptera also from the visual pathway (Farris and Sinakevitch, 2003).

Newly generated neurons in adult MBs derive from persisting MB neuroblast proliferation after development is concluded. Such ongoing production of new KCs occurs in house crickets and other coleopterans (Cayre et al., 1994), gryllidae (Cayre et al., 1996; Figure 1B), cockroaches (Gu et al., 1999), moths (Dufour and Gadenne, 2006), praying mantis, and milkweed bugs (Cayre et al., 2007). On the other hand, no active neuroblasts have been detected in MBs of honey bees (Fahrbach et al., 1995), ants (Gronenberg et al., 1996) and migratory locusts (Cayre et al., 1996). In *Drosophila*, neurogenesis in adult MB is scarce and was interpreted as last divisions of progeny, generated by neuroblast activity in the pupal stage (Ito and Hotta, 1992).

Integration of adult-born neurons into MBs is best understood in house crickets (*Acheta domesticus*), where life-long production of new interneurons is sustained by a cluster of approximately 100 neuroblasts located at the apex of a cortex formed by KCs (Cayre et al., 2007; Figure 1B). Asymmetric division of these neuroblasts leads to self-renewal and the production of a ganglion mother cell, which in turn divides symmetrically to generate two neurons. Newly formed KCs deposit in layers, whereby the most recent KCs are found close to the stem cells and older KCs, which are pushed away by new waves of KCs, settle in more peripheral layers (Cayre et al., 1996).

## New kenyon cells and olfactory learning?

Adult neurogenesis in crickets is substantial, accounting for an estimated 20% of total KCs in an aged cricket (Malaterre et al., 2002). KCs themselves are heterogeneous differing in size, projection and arborization patterns. In crickets, the adult-born KCs project all to the alpha and beta lobes of the MBs. In *Drosophila*, the alpha and beta lobes have been shown to be required for long-term memory formation (Pascual and Préat, 2001).

To understand the contribution of adult-born KCs in crickets, head-restricted gamma irradiation has been used to target adult dividing neuroblasts and study behavioral consequences. These experiments revealed that irradiated crickets showed slower learning in an olfactory association task and impaired long-term memory, whereas learning based on visual cues was mostly unaffected (Scotto-Lomassese et al., 2003). This suggests that new KCs could be important to link olfactory information processed in the antennal lobe to reward or danger signals (food, mates) in the environment. Such odor-reward integration leading to memory formation has been described in *Drosophila*, where olfactory learning leads to reinforcement in dopaminergic neurons innervating the MBs, which in turn drives synaptic plasticity between KCs and MB output neurons (Owald and Waddell, 2015). Hence reactivation of such remodeled MB circuits efficiently re-evokes odor-associated experience and guides appropriate approach or avoidance behavior.

In crickets, sensory stimulation alone is sufficient to increase neurogenesis (Cayre et al., 2005), but how selective memory is formed and retained is unclear, since all newly generated and pre-existing KCs survive. This is different in mammals where the bulk of adult-born neurons undergoes apoptosis (Biebl et al., 2000) and only few new neurons are selected to integrate into hippocampal circuits, which are structurally similar to MBs. It is therefore conceivable that new KCs may first serve to increase the acquisition rate in olfactory learning and then participate in olfactory-associative memories due to reinforcement occurring in specific subsets of KCs, but more functional experiments would be required to support this hypothesis.

In moths, adult-proliferating cells have been detected in MBs and optic lobes (Dufour and Gadenne, 2006). The brain of 3 days-old adults contained new KCs, but proliferation ceased shortly thereafter, when animals reached sexual maturity, which is similar in *Drosophila*. Moths are short-lived and males need to be able to locate females over large distances. This plasticity in the MBs could therefore be related to pheromone-associated learning to detect mates, but experimental evidence is lacking. Moreover, moths are able to associate olfactory cues such as pheromones or flower scents with a sugar reward and form long-term memory (Hartlieb et al., 1999), but learning has not been assessed in conditions of suppressed adult neurogenesis.

Possibly, the actual life span of an insect could be indicative if new neurons are generated in the mature brain. In short-lived insects, the production of new neurons in the adult brain may be too cost-intensive and time-consuming to yield an impact compared to species, which live up to 2 years (e.g. cockroaches). The analysis of more long-lived arthropod species such as spiders or termites, could shed some light on this question.

In the future, the red flour beetle may hold a great potential to uncover the function of neurogenesis in adult MBs since it combines the possibility to monitor adult neuroblast proliferation (lasting up to 2 months of adulthood) with the availability of genetic gain- and loss-of-function tools (Zhao X. et al., 2008).

## Insects: neurogenesis in sight?

Visual information in insects and crustaceans is processed in the optic lobe (OL) (Figure 1). In insects, the OLs are divided into the neuropils of the lamina, outer, and inner medulla and either lobular plate or a sublobula neuropil. In malacostracan crustaceans, there are four neuropils: the lamina, the medulla, the lobula, and lobular plate (Farris and Sinakevitch, 2003).

Several early histological studies mention the persistence of neuroblasts in adult OLs (Graichen, 1936; Panov, 1960). Proliferating cells verified by BrdU-labeling, have also been observed in OLs of adult moths (Dufour and Gadenne, 2006) and crickets (Cayre et al., 1996), but not further analyzed. In crickets, dividing cells were described to locate close to the medulla of the OLs (Scotto-Lomassese et al., 2003).

More recently, low-level adult neurogenesis has also been detected in the medulla cortex of *Drosophila* (Figure 1C), where a scattered population of neural precursors gives rise to new neurons (Fernández-Hernández et al., 2013; Moreno et al., 2015). These neural precursors do not express known neuroblast markers, except for cytoplasmic staining for Deadpan (Bier et al., 1992), a homolog of the vertebrate Hes (hairy and enhancer of split-1) bHLH transcription factors, which is expressed in the nucleus of dividing neuroblasts during development (Bello et al., 2008; Boone and Doe, 2008; Maurange et al., 2008). Lineage-tracing revealed that initial neural precursors and progeny divide mainly symmetrically to generate new neurons (Figure 1C). New-born neurons in the medulla cortex mature within 2 weeks, extending projections to lobula or lobular plate (Fernández-Hernández et al., 2013). In *Drosophila*, color-sensitive photoreceptors send their axons to the medulla, whereas light-dark sensitive receptors project to the lamina. The lobula is implicated in color and polarized light vision and spectral preference, the lobular plate is dedicated to motion detection (Behnia and Desplan, 2015).

Still, the proliferation of approximately 30–40 neural precursors per OL is very low given that they contribute to an estimated number of 4–6 divisions per OL per week, meaning that they are mainly quiescent. Based on the location and low activity of the neural progenitors, their function may be related to adjustments in color vision or slow homeostatic turnover.

Apart from neurogenesis, the optic neuropils have long been known to show volumetric plasticity (Heisenberg et al., 1995). If flies are reared in enriched environments (social interactions, light), the neuropils and especially the lamina become larger compared to animals deprived of light and housed alone. This effect is observable even in mature flies.

Workers of a species of nectar-feeding ants, naturally undergo a strong switch in behavior associated with visual input: They spend the first weeks of adulthood dedicated to tasks inside the nest before they set out to forage for food. This requires completely new abilities such as long-distance navigation and memory of food location. It would therefore be interesting to know if this transition is not only accompanied by growth of the optic neuropils and changes in photoreceptor expression (Yilmaz et al., 2016), but also by adult neurogenesis, especially given the context of long-distance navigation.

To conclude, given the scarce evidence on adult neurogenesis in the visual system, we can only speculate about its function. Notwithstanding the lack of data, the medulla seems to emerge as a potentially conserved site of adult neurogenesis in crustaceans and insects (Figure 1). To gain further insight into the role of adult neurogenesis in arthropods, it will be vital to conduct more functional experiments. The fact that *Drosophila* shows low-level adult neurogenesis and robust damage-induced formation of new neurons opens the door to address these processes with functional genetic experiments.

## The power of damage-responsive stem cells

**Figure 2:**
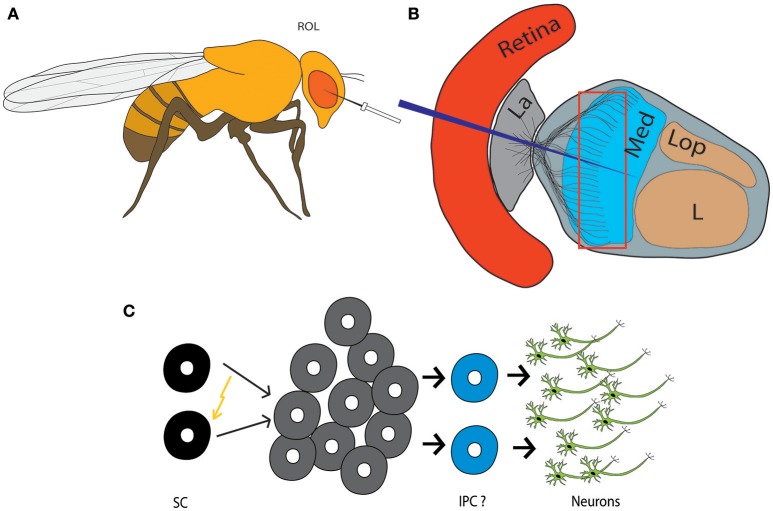
Injury-induced neurogenesis in the adult fly brain. **(A)** Traumatic brain injury paradigm in *Drosophila*: Stab lesions are performed with a thin metal filament to injure the right optic lobe (ROL). **(B)** Scheme depicting the retina and neuropils of the optic lobe. The eye is lesioned to the level of the medulla. La, Lamina; Med, Medulla; L, Lobula; Lop, Lobular plate. **(C)** Injury leads to activation and proliferation of quiescent adult neural progenitor cells, which give rise to new neurons, likely involving generation of a type of intermediate proliferating cell (IPC). SC, Stem cells.

Although the physiologic function of adult neurogenesis in *Drosophila* remains elusive, the adult neural progenitors in the OLs are readily activated in case of acute tissue damage. Stab lesion of the optic lobe to the level of the medulla (Figure 2), triggers proliferation of adult neural precursors, which involves nuclear translocation of Deadpan and upregulation of *Drosophila* Myc, the homolog of the human *c-myc* protooncogene (Fernández-Hernández et al., 2013). Such activated progenitors either directly or indirectly give rise to new neurons. The latter is more likely to be the case, since the damage-responsive precursors switch back to the resting state >48 h post-injury, but cells in proximity keep dividing (Fernández-Hernández et al., 2013; Fernández-Hernández and Rhiner, 2015). Stab wounding results in local regenerative neurogenesis around the lesioned site, which implies that neural progenitor cells distributed throughout the OL get either locally activated by injury-induced signals or may actively migrate to the site of damage. Remarkably, the newborn neurons mature and persist up to 11 days after injury (Moreno et al., 2015), which points to an efficient endogenous repair system. These findings underline that quiescent adult neural progenitors can serve as a backup population to regenerate brain tissue in case of injury.

In turn, the adult zebrafish brain contains numerous neurogenic regions, which harbor neural progenitors (radial glial cells) that constantly produce neurons in the mature brain (Kizil et al., 2012). Upon stab lesion of the telencephalon, inflammatory signals stimulate radial glial cells to increase proliferation and the damaged brain area is efficiently restored (Kyritsis et al., 2012). Teleost fish show therefore a trend that continuous brain growth during adult life is paralleled by pronounced adult neurogenesis, similar to crustaceans.

A promising therapeutic strategy aims to support local neurogenesis in human patients suffering from trauma or stroke, circumventing the complications of grafting exogenous stem cells for repair. Endogenous adult neural stem cells have been shown to be similarly responsive to injury signals in rodents and humans (Zhang and Chopp, 2009): After stroke, neural stem cells in the SVZ enhance proliferation, reactivate developmental pathways and generate neuroblasts, able to migrate to lesioned brain areas (Zhang and Chopp, 2009). In addition, a large fraction of neuroblasts are produced locally at the ischemic lesion from activated astrocytes, which adopt a neurogenic program (Magnusson et al., 2014). Major challenges are however the survival and successful integration of the newly formed neurons and the tendency of the neural stem cells to form glial instead of neural cells.

The above-described model of brain regeneration in the genetic model organism *Drosophila* allows to screen for conserved genes, which are important for the efficient activation of quiescent neural progenitors and their differentiation into neural lineages *in vivo*. Moreover, the newly-generated cells can be followed up to study how they integrate into circuits. Initially, optic lobe lesions should allow to evaluate the effect of regenerative neurogenesis on recovery of visual skills. Learnt principles may then be tested in other contexts, such as ventral nerve cord regeneration in flies (Kato et al., 2011), which are relevant for human CNS injuries.

So far, crustaceans and insects have played a very small role in the field of adult neurogenesis, which has been dominated by mammalian research. Recent studies have uncovered fascinating aspects of stem cell regulation and regeneration in arthropods, which may open new therapeutic approaches to stimulate neurogenesis after brain injury.

## Author contributions

ARS and CR wrote the manuscript. CR elaborated the concept and ARS drew the Figures.

### Conflict of interest statement

The authors declare that the research was conducted in the absence of any commercial or financial relationships that could be construed as a potential conflict of interest.
